# Outcomes following autologous tumor tissue implantation with or without concurrent antineoplastic therapies in the treatment of sarcoids in 50 equids

**DOI:** 10.3389/fvets.2025.1559519

**Published:** 2025-05-09

**Authors:** Caitlin H. Smith, Holly L. Stewart, Darko Stefanovski, David G. Levine

**Affiliations:** Department of Clinical Studies, New Bolton Center, University of Pennsylvania, Philadelphia, PA, United States

**Keywords:** sarcoid, autologous, autoimplantation, regression, vaccine

## Abstract

This study aimed to describe the effect of single implantation of autologous tumor tissue at inducing clinical regression of equine sarcoids. Special emphasis was placed on the influence of concurrent and subsequent therapies, time- independent outcomes, and associated complications. A retrospective review was conducted using medical records from the University of Pennsylvania’s New Bolton Center between May 2014 and January 2022. Follow-up data were collected through phone and email surveys. Descriptive statistics were generated, and outcomes were analyzed using univariate and multivariate logistic regression models. Fifty equids that underwent elective autologous tumor tissue implantation were included in the study. Complete resolution without recurrence was observed in 50% of cases. There was no significant difference in outcome between animals treated with autoimplantation alone and those receiving concurrent antineoplastic therapies. Equids with a history of treatment failure were 77% less likely to show improvement. After adjusting for other factors, sarcoids located on the body had 48% lower odds of clinical improvement, and each additional tumor decreased the odds of improvement by 11%. When tumor numbers decreased following initial implantation, the odds of recurrence were reduced by 71%. Two cases (4%) developed tumors at the implantation site. Autologous tumor implantation was most effective in animals with a lower tumor burden and was relatively less successful in cases involving body-localized sarcoids or tumors refractory to previous treatments. Severe complications were uncommon, and client satisfaction trended with incidence of recurrence. The technique is technically simple and may be beneficial in selected cases of equine sarcoids. Further research into the mechanisms may inform the development of future therapies, including potential commercial vaccines.

## Introduction

Sarcoids are equid-specific, fibroblastic dermal growths that typically do not metastasize and display a predilection for sites on the head, body, and paragential regions (1). The locally expansile and recurrent nature of the tumors can lead to welfare and performance problems in affected animals. Bovine papilloma virus (BPV) types 1, 2 and 13 have been consistently linked to the development of sarcoids in horses, although clinical behavior of the sarcoid-associated virus is dissimilar to that found in cattle (2–6). The departure from normal viral behavior may contribute to reduced activation of cell-mediated and humoral immune responses as found with other infections. It is theorized that infection is propagated via vector or direct transmission, though exact infection pathogenesis has not been established (7). A consistently effective treatment has been not identified to definitively treat sarcoids, and multimodal approaches have been often demonstrated to be the most successful at achieving resolution (8–10). Historically employed treatments include surgical excision (8, 11), cryotherapy (8, 9, 12), laser surgery (9, 13), radiation therapy (11), electrochemotherapy (14, 15), systemic vaccination (16–18) and local immune modulation (10–13, 17, 19–29). Reported success is variable, and recurrence, often hyperproliferative, is common (2, 9). Several factors have been implicated in the recurrent nature of sarcoid tumors including animal age, sex, genetic predisposition (2), and tumor histopathology or phenotypic characteristics (6, 30).

Several attempts have been made to develop a vaccine to target the viral origins of the disease (16–18). There are several reports of autologous vaccinations being created from tumor tissue components for papillomavirus associated infections (31, 32), though the underlying mechanism of action of autoimplantation is currently unknown. It is hypothesized that removal, treatment and re-introduction of the viral-laden tumor tissue allows the adaptive immunities to mount an appropriate systemic anti-viral response that prevents recurrence of the disease following excision. A study evaluating autoimplantation of anal and genital warts associated with human papilloma virus found a 26% regression rate, 44% cure rate and 0% recurrence of disease in cured cases (33). A previous retrospective analysis evaluated 18 horses with sarcoids that were treated with autoimplantation and reported a decrease in the number of tumors in 75% of horses, a decrease in the size of tumors in 93.8%, and complete resolution of disease in 69% of horses (34). Given the positive preliminary data for autoimplantation, the investigators were motivated to perform a larger-scale analysis on horses receiving autologous implantation in combination with other antitumoral therapies to understand whether this was an effective treatment for sarcoids.

The objective of this study was to evaluate whether sarcoid autoimplantation can induce regression in horses seen at a tertiary referral hospital. It was hypothesized that (1) a single autoimplantation treatment would be sufficient to induce sarcoid regression in adult horses, and (2) the addition of concurrent immunomodulating therapies would improve outcomes relative to the previously published results. Outcomes from this study were focused on describing whether clinical improvement occurred regardless of the time course to occurrence. The reported data was intended to help clinicians and owners evaluate available treatment protocols for affected animals.

## Methods

### Case selection

Medical records from the University of Pennsylvania’s New Bolton Center were reviewed from May 2014 to January 2022. Animals of any age and breed that underwent elective sarcoid tumor resection and autologous implantation in the hospital were included. All animals were required to have histopathologic confirmation of sarcoid diagnosis prior to implantation. If animals underwent two implantation procedures, the second episode was recorded as a subsequent treatment. Previously described cases (34) were excluded. Cases that underwent tumor resection or other treatment without implantation were excluded.

### Data collection

Retrospective data was collected from each record including animal signalment (age, sex, breed), tumor characteristics (number of tumors, total tumor area (cm^2^)), anatomic location, historical treatments performed, mitotic count reported in histopathology report, and prescribed perioperative mediations. Breed and anatomic location (head, neck/chest, body, limb) of tumor were recorded categorically. Sarcoids reported on the axilla, ventrum, and parainguinal regions were grouped with “body.” Tumor area was calculated using the largest dimensions provided in the medical record if the area was not explicitly recorded. Tumor number was treated as a continuous variable in analysis with categorical groupings provided for preliminary evaluation.

### Surgical procedure

All autologous implantation procedures were performed as previously described (34). Briefly, the patient was either sedated and restrained standing or induced under general anesthesia as necessary to facilitate resection of the sarcoid depending on anatomic location and patient demeanor. Medication protocols varied depending on clinician or anesthetist preferences. The sarcoid tumor(s) to be removed and the surrounding skin were prepared by clipping, cleaning with 4% chlorhexidine gluconate and rinsing with clean water. Subcutaneous perilesional infiltration of 2% mepivacaine (Carbocaine-V; Zoetis US) was administered in all standing cases. The lesion was then debulked to the level of the skin, leaving the base *in situ*, and any resulting hemorrhage was controlled by manual compression with sterile gauze. The removed sarcoid tissue was cleaned of any gross necrotic debris and sectioned into several small cubes measuring about 0.5 cm x 0.5 cm x 0.5 cm. The pieces were placed in sterile gauze and submerged fully in liquid nitrogen for 10 min. The instruments used to resect and section the tumor tissue were discarded, and the surgeon’s gloves were changed.

For implantation of the treated tissue, an area immediately ventral to the nuchal ligament, 10 cm long and 5 cm wide, was clipped and aseptically prepared. Mepivicaine hydrochloride 2% (2–3 mL each) was infused subcutaneously in 2 to 3 locations within the prepared area. After 10 min, the treated sarcoid tissue was lifted from the liquid nitrogen and rinsed with warm saline. Stab incisions were created through the skin and subcutis using a new number 10 scalpel blade. Hemostatic forceps were used place 2 to 4 cubes of sarcoid tissue into each stab incision. The skin was closed over the implanted sarcoid with 1 to 2 simple interrupted or cruciate sutures with either 2–0 polypropylene or 2–0 poliglecaprone 25 suture dependent on clinician preference. Aerosol, aluminum bandage (AluSpray® Aluminum Powder; Neogen®Vet) was applied to the resection and implantation sites. Perioperative non-steroidal inflammatory medications, antibiotics and additional immunomodulating therapies were prescribed at the clinician’s discretion.

### Follow up

Standardized phone and email surveys were used to collect follow-up data including complications in the immediate postoperative period, response of the sarcoid(s) to autologous implantation, incidence of recurrence, subsequent treatments performed, and client satisfaction. Gross changes in tumor parameters following treatment were subjectively reported, and validation of client description of lesions was confirmed with veterinary evaluation when possible. Tumor response was reported as “clinically improved” if there was any subjective reduction in tumor number or total tumor size (cm^2^) according to client communication following autologous implantation. Tumor response was further considered “resolved” if the animal was reported to be macroscopically free of tumors at any time following implantation and before any subsequent treatments. Tumors were described as “recurrent” if any increase in number or total size of tumors was observed following clinical improvement or resolution. Timeline of subsequent therapies performed following recurrence was estimated by clients or confirmed in medical records when applicable. Client satisfaction was assessed with a binomial yes or no question. If dissatisfaction was reported, respondents were asked to provide further clarification.

### Statistical analysis

All data were collected and stored in a Microsoft Excel worksheet for analysis. Categorical variables were reported as frequency count (percentage of total), and continuous variables were assessed for normality using the Shapiro–Wilk test and visual evaluation of histograms. All descriptive continuous data were non-parametric and reported median and range.

Univariable logistic regression was used to assess the association between independent, explanatory variables and outcomes. Mixed effect logistic regression was performed, with horse as a random effect. Outcomes of interest were initial regression of sarcoid in response to treatment, and sarcoid recurrence following autologous treatment. Multicollinearity was checked using the variance inflation factor and R^2^ for each individual independent variable. Independent variables with evidence of association (defined as *p* ≤ 0.2) in univariable logistic regression analysis were selected for multivariable logistic regression analysis. Model fit was assessed using regression evaluation metrics, including Tjur R^2^ and Akaike information criterion (AIC). Higher values for Tjur R^2^ and lower AIC values represented better model fit. The final model was constructed using stepwise backward selection to remove non-significant independent variables from the model. Variables were considered significantly associated with the outcome of sarcoid regression with autologous treatment when *p* < 0.05. Uni- and multivariate regression models are described as odds ratios (ORs) and 95% confidence intervals (CIs). *p* < 0.05 was used to determine statistical significance. All data were analyzed using R software (version 4.2.2, R Foundation of Statistical Computing 2022) in RStudio (version 2022.12.0 + 353) and GraphPad Prism (version 10.3.0, GraphPad Software Inc).

## Results

### Sample population

A total of 64 clinical records were selected for review, and follow-up was attained for 50 animals. The 14 cases lost to follow-up were not included in any statistical analysis. Median time between autologous implantation procedure and follow up data collection was 213 weeks (range: 46–507 weeks). The sample population breed and sex distributions are included in Table 1. The median age of animals was 11 years (2–25 years).

**Table 1:** Univariate logistic regression model describing the association between the explanatory variables and clinical improvement of sarcoid tumors (outcome) in response to implantation of autologous tumor tissue.

Explanatory variable	Category	N, % of population	Odds ratio	95% confidence interval	*p-v*alue
Age (years)	-	-	0.934	0.833 to 1.038	0.213
Breed	Warmblood (Reference)	19/50, 38%	-	-	0.087^*^
	Draft/Draft crossbreed	2/50, 4%	1.693	0.843 to 3.397	0.135
	Donkey	1/50, 2%	1.693	0.647 to 4.428	0.276
	Quarter Horse^b^	14/50, 28%	0.956	0.687 to 1.330	0.784
	Pony/Pony crossbreed	2/50, 4%	1.693	0.843 to 3.397	0.135
	Standardbred	1/50, 2%	0.623	0.238 to 1.630	0.326
	Arabian	1/50, 2%	1.693	0.647 to 4.428	0.276
	Thoroughbred	10/50, 20%	1.532	1.062 to 2.209	0.024
Sex	Mare (Reference)	22/50, 44%	-	-	0.207^*^
	Gelding	28/50, 56%	0.762	0.236 to 2.386	0.642
Anatomic location^a^	Limb (Reference)	4/50, 8%	-	-	0.086^*^
	Head	19/50, 38%	0.729	0.430 to 1.237	0.235
	Neck	12/50, 24%	0.659	0.379 to 1.147	0.137
	Body	14/50, 28%	0.526	0.305 to 0.906	0.022
Number of tumors	-	-	0.630	0.413 to 0.923	0.022
Total tumor area (cm^2^)	-	-	0.996	0.993 to 1.000	0.042
Historical treatment performed	No (Reference)	38/50, 76%	-	-	0.054^*^
	Yes	12/50, 24%	0.238	0.0525 to 0.940	0.047
Mitotic count (/hpf)	-	-	0.984	0.933 to 1.022	0.421
Imiquimod prescribed at time of procedure	No (Reference)	25/50, 50%	-	-	0.232^*^
	Yes	25/50, 50%	0.591	0.159 to 2.065	0.416
Cisplatin administered at time of procedure	No (Reference)	47/50, 94%	-	-	0.528^*^
	Yes	3/50, 6%	1.636	0.145 to 36.87	0.697
EqStim^c^ administered at time of procedure	No (Reference)	46/50, 92%	-	-	0.143^*^
	Yes	5/50, 8%	0.643	0.0718 to 5.746	0.672
NSAID (days of administration)	-	-	0.030	−0.048 to 0.108	0.438

* Overall *p* value. Hpf = high powered field.

a Reported anatomic location of sarcoid tumor(s). Sarcoids reported on the axilla, ventrum, and parainguinal regions were grouped with “body,” and sarcoids reported on the chest were included in “neck.”

b Quarter Horse classification included Paint and Appaloosa.

c EqStim Propionibacterium Acnes Immunostimulant; Neogen Vet.

### Lesion characteristics

The median reported tumor area was 16 cm^2^ (range: 2–250 cm^2^). The distribution of number of individual sarcoid tumors was categorized as 1 lesion (25/50, 50%), 2 lesions (10/50, 20%), 3–4 lesions (7/50, 14%), and 5 or more lesions (8/50, 16%). The distribution of anatomic location of tumors is included in Table 1.

Historical treatment of sarcoid tumors prior to autologous implantation was reported in 12/50 cases (24%), and four cases (4/12, 33%) underwent multiple historical treatments. Reported historical treatments included topical medications (*n* = 7), surgical resection or debulking (*n* = 4), intralesional cisplatin (*n* = 3), electrochemotherapy (*n* = 1).

A preoperative histopathological diagnosis of sarcoid was confirmed in all cases. Histopathology reports were available for review in 33/50 (66%) of cases. For 17 cases, only the histopathological diagnosis, but not the detailed description of biopsy findings, was present in the medical record at the time of review. The mitotic count was recorded as “rare” in 18/33 (55%) of available reports. In the 15 reports with numerical values reported, the median mitotic count was 0.5/10hpf (range: 0–9/10hpf).

### Perioperative variables

The majority of autoimplantation procedures were performed in standing sedated horses (38/50, 76%). The remaining 24% of procedures were performed under general anesthesia in a surgical suite. Non-steroidal anti-inflammatory drugs were administered in 42/50 cases (84%) for a median of 4 days (1–9 days). Antibiotics (trimethoprim-sulfamethoxazole) were prescribed in 6 cases (12%). The distribution of cases treated with concurrent antitumoral medications is included in Table 1. Complications in the immediate postoperative period were reported in 14/50 cases (28%). Reported complications included incisional infection at either tumor resection or implantation sites (*n* = 6), dehiscence of tumor implantation site (*n* = 4), adverse reactions to imiquimod (*n* = 4), excessive scarring (*n* = 1), and prolonged hemorrhage (*n* = 1). One case reported two types of complications (incisional infection and reaction to imiquimod).

### Clinical response

Complete resolution of sarcoid tumors following a single autologous implantation procedure without recurrence was reported in 25/50 cases (50%). Owner description of tumor response was confirmed by a veterinarian in 15/50 cases (30%). The univariate logistic regression between the explanatory variables recorded and tumor response is shown in Table 1. Based on these results, a multivariable model was constructed to include anatomic location, number of tumors, total tumor area (cm^2^) and historical treatment (Table 2). Thoroughbreds demonstrated perfect collinearity with outcome, and thus breed was excluded from the multivariate regression model.

**Table 2:** Multivariate logistic regression model describing the association between the independent variables and clinical improvement of sarcoid tumors (outcome) in response to implantation of autologous tumor tissue.

Independent variable	Category	Odds ratio	95% confidence interval	*p*-value
Anatomic location^a^	Limb (Reference)	-	-	0.024^*^
	Head	0.655	0.398 to 1.078	0.093
	Neck	0.823	0.475 to 1.428	0.478
	Body	0.523	0.319 to 0.855	0.011
Number of tumors	-	0.890	0.813 to 0.984	0.024

* Overall *p* value.

a Reported anatomic location of sarcoid tumor(s). Sarcoids reported on the axilla, ventrum, and parainguinal regions were grouped with “body,” and sarcoids reported on the chest were included in “neck.”

### Recurrence

Recurrence of sarcoid tumors following implantation occurred in 24/50 cases (48%). In one case, the sarcoids were reported to partially improve following autoimplantation and then remained unchanged until the time of follow up. Of cases in which tumors had been reported to completely resolve, 5/30 (16%) recurred. Of the 20 cases of tumors which had a reduction in size or number following implantation but did not completely resolve, 19 (95%) progressed (included in recurrence). Of horses that had 5 or more tumors initially reported, 7/8 (87.5%) reported recurrent disease. Of the horses that had historical treatments recorded, 10/12 (83%) reported recurrent disease. The univariate logistic regression between the explanatory variables recorded and recurrence is shown in Table 3. Decrease in tumor number (R^2^ = 0.276) and decrease in tumor size (cm^2^, R^2^ = 0.065) were utilized for the multivariable logistic regression analysis (Table 4). A decrease in the number of tumors (*p* = 0.010), but not a decrease in the size of the tumor tissue (*p* = 0.662), was significant when predicting whether the tumors would recur following implantation of autologous tissue.

**Table 3:** Univariate logistic regression model describing the association between the explanatory variables and recurrence of sarcoid tumors (outcome) in response to implantation of autologous tumor tissue.

Explanatory variable	Category	Odds ratio	95% confidence interval	*p*-value
Decrease in number of tumors following implantation	No (Reference)	-	-	0.017^*^
	Yes	25	4.200 to 482.8	0.003
Decrease in size of tumors (cm^2^) following implantation	No (Reference)	-	-	0.159^*^
-	Yes	5.776	0.8842 to 113.5	0.117

* Overall *p*-value.

**Table 4:** Multivariate logistic regression model describing the association between the independent variables and recurrence of sarcoid tumors (outcome) in response to implantation of autologous tumor tissue.

Independent variable	Odds ratio	95% confidence interval	*p*-value
Decrease in number of tumors following implantation	33.92	3.87 to 1,165	0.010
Decrease in size of tumors (cm^2^) following implantation	0.529	0.020 to 14.20	0.662

Twenty of the sarcoid tumors that recurred (83%) occurred in the same location as previously reported. Four cases (17%) reported growth of tumors in locations different than originally described including two cases (4%) with growth of tumors at the implantation site. No cases with recurrent tumors in the same or new anatomic locations had subsequent biopsy or histopathology performed.

### Subsequent treatment

Of the cases that reported recurrent disease, 17/24 (70%) received subsequent treatment following recurrence. Time to subsequent treatment was reported for 15/17 cases, and median time to subsequent treatment was 24 weeks (range: 12–88 weeks). Nine cases (9/17, 53%) had two or more types of subsequent treatments performed. Subsequent treatments included surgical debulking (*n* = 9), topical medications (*n* = 5), intralesional cisplatin (*n* = 4), immunostimulatory injections (*n* = 4), repeated autologous implantation (*n* = 2), electrochemotherapy (*n* = 1), and radiation therapy (*n* = 1). Of the cases that received subsequent treatment, 8/17 (47%) reported secondary clinical improvement. Of the cases that had historical treatment recorded, underwent autoimplantation and received subsequent treatment, 3/7 (42%) reported secondary clinical improvement.

### Client satisfaction

Of the clients interviewed, 39/50 (78%) reported being satisfied with the results of the autologous implantation procedure. Recurrence was the primary reason for dissatisfaction among owners (10/11, 91%). Other reasons cited for dissatisfaction included incisional complications and prolonged duration between surgery and regression.

## Discussion

Single implantation of autologous tumor tissue induced resolution of sarcoids without recurrence in 50% of cases at the time of follow-up. No significant differences were observed in animals receiving concurrent immunomodulatory or antitumoral therapies compared to those without. These findings are consistent with the success rates previously described by Rothacker et al. (34) and are comparable to other treatment modalities including excisional techniques (plain surgical excision: 69.8–78% (35), laser excision: 71% (8)), electrochemotherapy: 44% (1, 4), cryotherapy: 9–79% (8, 11, 12), radiation therapy: 98% (11), local immunotherapies (imiquimod: 80–84.4% (10, 25), 5-fluorouracil: 61.5% (23), BCG vaccination: 67–69% (8, 11), IL-2: 36–50% (24), cisplatin: 83–98% (15), cisplatin + IL-2: 80% (24), and acyclovir: 9–100% (27, 28)) or topical homeopathic remedies (*Viscum album austriacus*: 67% (20) and *Sanguinaria canadensis*: 89.2% (10)). Direct comparisons with much of the available body of literature are challenging due to variations in tumor responses reported and evaluation methods. Moreover, many of the referenced studies use serial or prolonged treatment courses, whereas this report evaluates outcomes following a singular autoimplantation event.

The demographic distributions identified in the current study reflect those described in the literature. Sarcoids occur in equids of any mature age and have a slight predilection towards males (7, 9, 29). Warmbloods, Quarter Horses and Thoroughbreds were the most commonly represented breeds, while Standardbreds and Arabians demonstrated lower incidence (1, 9, 22, 30). The significant association of Thoroughbred with clinical response in this study was most likely due to a low sample population rather than unanimously favorable response by the breed.

Tumor location distribution was consistent with previous reports with head, neck and body, namely the parainguinal region, being most frequently affected (7, 24, 29, 30). All available preoperative histopathology reports supported diagnosis of equine sarcoid as described in the literature (6, 36). Although an increased recurrence rate of sarcoid tumors was linked to mitotic count of ≥20/10 hpf (30), no association between mitotic index and regression or recurrence was observed in the current study. This discrepancy could be explained by the incomplete number of detailed biopsy reports available, and the absences of mitotic counts greater than 9/10 hpf.

Factors influencing clinical improvement following autoimplantion included total tumor number, size, location on the body, and prior treatment history. These trends were consistent with previous reports (8, 9, 23, 37). Smaller tumor number and size represented less severe disease which had generally improved outcomes. Regression analysis identified sarcoid location as a significant variable, but only when limb was used as the referent category. Without a gold standard referent cited in the literature, regression analysis was performed with all sarcoid locations as the referent category. Given this, the conclusion that sarcoids located on the body have a lesser odds of regression following autoimplantation should be interpreted cautiously especially given the low numbers of sarcoids reported in this location and across all locations in the study.

The influence of historical treatment on clinical outcomes remains inconclusive. Anecdotal reports suggest aggressive recurrence of sarcoids following incomplete treatment, but there is no evidence that the tumors become more resistant following therapy (21). Histopathologic evaluations of primary and recurrent tumors have not found meaningful differences in their composition (36). This may suggest that difficulties in treating persistent tumors result from the virus-immune system interaction rather than tumor composition, which further supports the use of a vaccine-based treatment like autoimplanted tumor tissue.

The recurrence rate reported in the current study (48%) parallels the rates of recurrence of other treatment modalities ranging from 7.3–91% (8, 10–12, 23, 30). While the persistence of any tumor following autoimplantation suggests treatment failure, a reduction in absolute tumor number was found to be a more reliable predictor of recurrence than change in tumor size. The ability of autoimplantation to treat several tumor sites simultaneously highlights the advantage of systemic treatments over focal therapies. The overall higher recurrence rate identified in this study compared to that reported by Rothacker et al. (34) may be due to the larger sample population and/or the extended time to follow up. For prognostication purposes, it can be inferred that recurrence may be expected within 24 weeks of treatment (the median time reported from autoimplantation to subsequent treatments).

Development of tumors at the site of autologous implantation has been recounted anecdotally but is not currently reported in the literature. In the present study, the two affected cases had implantation procedures performed within 4 weeks of each other, but no other similarities in case presentation, lesion description or progression were identified. The tumor mass was macroscopically evaluated by the primary veterinarian and diagnosed as sarcoid in one of the two cases. Gross appearance and historical behavior alone are 82% accurate in diagnosing equine sarcoid (38). The tumor in this case resolved with laser resection and had not recurred at the time of follow up. The other case failed topical treatment with *Sanguinaria canadensis*, and the tumor was present on the neck at the time of follow up. Development of sarcoid tumors at the site of implantation could be due to surgical contamination during the tissue handling process or incomplete viral denaturation (39). This phenomenon is uncommon as special care is taken to avoid seeding tumor tissue using strict no touch surgical techniques.

Although no significant benefit was identified with the addition of concurrent immunotherapies, existing literature supports the use of multimodal protocols to treat sarcoids (8, 9, 29). Autoimplantation is hypothesized to attenuate the adaptive immune response against BPV, while concurrent local treatment recruits the innate immune response to eliminate persistent viral infection. The necessity of adjunctive therapies in addition to systemic vaccination likely reflects the local immune-evading environment created by sarcoids (2, 40–42). Application of topical immunomodulatory medications or subsequent excision following autoimplantation may enhance outcomes by physically removing residual tumor tissue and inducing superficial inflammation at the application site which has been associated with reduced recurrence (43, 44). Secondary clinical improvement was observed in almost half of cases (47%) with recurrence suggesting that autoimplantation can induce longer lasting changes to the immune environment. A similar incidence of secondary improvement was observed in both historically treated and untreated sarcoids, possibly providing a solution to persistent tumors that do not respond to local treatment alone.

The limitations of the current study include its retrospective nature, incomplete medical records, and lack of follow up information for a large proportion of cases (14/64, 22%). The results are likely underpowered, and a prolonged follow up period may have influenced outcomes. The authors were motivated to examine the interaction of autoimplantation with concurrent therapies so analysis of time-to-event data was not reported within the scope of this study but should be included in analyses of future works. The lack of control data available for comparison and the potential selection bias of the patient cohort likely also restricts the generalizability of the conclusions. Additionally, the reliance on client-reported observations of tumor response introduces uncertainty since the accuracy of most reports was not confirmed by veterinary evaluation. Changes in size or tumor number were evaluated qualitatively, and microscopic confirmation of resolution was not performed.

Standardized treatment protocols can be improved upon in future studies to minimize unanticipated variability of clinical responses. Phenotypic categorization was not available for regression modeling in this report, but inclusion is warranted since phenotype has been correlated with clinical behavior and varying viral loads (5, 6, 30). While the genetic components of BPV have been consistently isolated in all sarcoid phenotypes (13, 14), it is possible that disparities in distribution of viral components could affect the clinical outcomes (45). Furthermore, more rigorous evaluation of tumor tissue following submergence in liquid nitrogen is required to confirm appropriate inactivation of viral components. The influence of tissue segment size, duration of exposure or repeated freeze–thaw cycles are currently unknown. Future studies should aim to improve upon these limitations and explore the pathophysiology of the underlying immunologic mechanisms. Prospective evaluation of systemic immune markers could be an opportunity to compare the effects of autologous implantation to standardized vaccinations. Significant increases in adaptive immunologic parameters, specifically systemic T-lymphocyte concentrations and circulating IgG molecules, were reported following human autoimplantation procedures (33), and increased circulating IgG were identified after administration of other sarcoid vaccine formulations in horses.

To summarize, autologous implantation of sarcoid tissue is a promising treatment approach that may exploit the viral characteristics of the disease. The systemic treatment provides acceptable single procedure results and may encourage long-term resolution. While the addition of concurrent therapies does not significantly enhance outcomes, the rate of success continues to produce satisfactory results for clients.

## Data Availability

The raw data supporting the conclusions of this article will be made available by the authors, without undue reservation.
